# Living with early-onset type 2 diabetes in young adulthood: a narrative review of lived experience and implications for developmentally appropriate care

**DOI:** 10.3389/fendo.2026.1903159

**Published:** 2026-09-22

**Authors:** Joel Song Rui Goh, Li Xuan Sim, Ian W. Seetho, Shih Ling Kao, Run Ting Chin, Chin Meng Khoo, Amanda Yuan Ling Lim, Pei Chia Eng

**Affiliations:** 1 Lee Kong Chian School of Medicine, Nanyang Technological University, Singapore, Singapore; 2 Division of Endocrinology, Department of Medicine, National University Hospital, Singapore, Singapore; 3 Department of Metabolism, Digestion and Reproduction, Imperial College London, London, United Kingdom

**Keywords:** developmentally appropriate care, early-onset type 2 diabetes mellitus, lived experience, self-management, stigma, young adulthood

## Abstract

**Introduction:**

Early-onset Type 2 Diabetes Mellitus (T2DM), defined as diabetes diagnosis before 40 years old, is associated with substantial lifetime cardiometabolic risk. Although many challenges of living with T2DM are shared across adulthood, diagnosis during young adulthood may shape how this condition is perceived, prioritised and managed during a formative stage of life. This narrative review aimed to synthesize how adults diagnosed with T2DM between 18 and 39 years of age experience, understand and manage their condition, and the implications for developmentally appropriate clinical care.

**Methods:**

We conducted a structured narrative review of English-language qualitative, mixed-methods and observational quantitative studies identified in PubMed, MEDLINE (via Ovid) and EMBASE (via Ovid) from database inception to 31 August 2025. Direct evidence from adults diagnosed at 18 to 39 years was prioritised. Studies of adults aged ≥18 years with youth-onset T2DM were used as closely related supporting evidence, and broader adult T2DM studies as contextual evidence. Study selection, extraction and appraisal followed predefined procedures, with reporting informed by SANRA.

**Results:**

Recurring themes included difficulty integrating diabetes into identity, stigma, competing work and family demands, material hardships, family and sociocultural influences and healthcare services perceived as not aligned with young adults’ priorities. Young adults valued practical and age-appropriate communication, flexible appointment scheduling, credible digital education and peer support. However, much of this evidence was cross-sectional or derived from small, single-site qualitative studies and intervention evidence was limited.

**Conclusion:**

Although many challenges overlap with the broader T2DM population, they may have particular significance when diabetes develops during young adulthood. Developmentally appropriate care should combine cardiometabolic risk management with flexible, collaborative and non-stigmatizing support. Prospective studies are needed to test whether such models improve patient-reported, clinical and health-service outcomes.

## 1 Introduction

Worldwide prevalence of type 2 diabetes mellitus (T2DM) has risen substantially in recent years and is projected to reach 853 million cases by 2050 (1). Early-onset T2DM, defined as diagnosis of diabetes in individuals before the age of 40 (2), is particularly concerning: incidence has increased two- to threefold since 2000, posing significant public health challenges. Compared with people without diabetes, individuals with early-onset T2DM face at least a twofold excess mortality risk, which increases in a log-linear manner with younger age of diagnosis (3). Early-onset T2DM is also associated with a more aggressive disease phenotype than later-onset T2DM, characterized by a faster β-cell decline (4), higher glycated hemoglobin (HbA1C), higher body mass index (BMI), a more adverse cardiometabolic risk factor profile (5), earlier insulin dependence (6) and a greater risk of both microvascular and macrovascular complications (4).

Early and intensive management of T2DM may reduce diabetes-related complications (7), yet clinical guidance for young adults remains limited. Early-onset T2DM is commonly defined as diagnosis before 40 years of age but published studies use heterogenous age thresholds and often combine youth-onset (<18 years old) and early adult diagnoses (8). This review focuses on young adults diagnosed between 18 and 39 years of age, while acknowledging studies using closely related upper thresholds where the population was explicitly described as young- or early-onset T2DM. This distinction is important because diagnosis during young adulthood coincides with workforce entry, increasing independence, family formation and reproductive planning (9). Young adults with T2DM report greater diabetes-related distress (10), lower adherence to treatment (11) and higher levels of stigma (2) than those with later-onset T2DM. Despite this, healthcare delivery for young adults with early-onset T2DM remains largely inflexible and poorly attuned to psychological, social and practical realities of life stages.

This narrative review aimed to synthesise evidence on how young adults diagnosed with T2DM between 18 and 39 years of age experience, understand and manage their condition, and to identify barriers to diabetes care and the implications for developmentally appropriate clinical care. The review question was: How do young adults with early-onset T2DM perceive and experience their condition, self-management and healthcare, and what implications do these experiences have for the design of developmentally appropriate services? A narrative approach was selected because the evidence is methodologically heterogeneous, with variation in age definitions, populations, study designs and outcomes. The intention was to provide a critical thematic synthesis rather than quantitative pooling.

## 2 Methods

We conducted a narrative literature review of qualitative, mixed-methods and observational quantitative studies examining the experiences of young adults living with early-onset T2DM. Although this was not designed as a systematic review, we adopted structured search, screening, extraction and critical-appraisal procedures for transparency and reproducibility. Reporting was strengthened with reference to the Scale for the Assessment of Narrative Review Articles (SANRA). PubMed, MEDLINE (via Ovid) and Embase (via Ovid) were searched from database inception to 31 August 2025. Search concepts combined terms relating to T2DM, early- or young-onset disease/young adulthood and lived experience, perceptions, beliefs, stigma, distress, self-management, healthcare and support. The complete database-specific search strategies, including controlled vocabulary where applicable, free-text terms, Boolean operators and field restrictions, are provided in **Supplementary Table S1a**. Searches were restricted to English-language publications. Reference lists of included studies and relevant reviews were also examined to identify additional eligible studies.

Duplicate records were removed before screening. Two reviewers independently screened the retrieved records by title and abstract against predefined eligibility criteria. Potentially relevant articles were subsequently assessed at full text by the same two reviewers. A third reviewer independently checked the extracted information against the source articles; discrepancies were resolved through discussion, with consultation from a fourth reviewer where required. For each included study, we extracted author and year, country, study design, sample characteristics and sample size, age range and definition of young- or early-onset T2DM, population applicability to the review question, experiential or outcome domains examined, principal findings and key methodological limitations. Studies were additionally classified as direct, closely related supporting or contextual evidence according to their applicability to adults diagnosed with T2DM between 18 and 39 years of age. The numbers of records identified, screened, assessed at full text and included in the final synthesis are reported in the study-selection flow diagram (**Supplementary Figure 1**).

### 2.1 Eligibility criteria

We prioritized original studies involving adults aged 18 years and above who were diagnosed with T2DM before 40 years of age, or populations explicitly described as young- or early-onset T2DM. Qualitative, mixed-methods and observational quantitative studies were eligible when they reported patient perspectives or psychosocial, behavioral, social or healthcare-experience outcomes. Studies focused exclusively on T1DM, gestational diabetes, participants younger than 18 years old or mixed diabetes populations without separately extractable T2DM findings were excluded. Studies involving young adults aged ≥18 years who had developed T2DM during childhood or adolescence were not considered direct evidence for adult-onset early T2DM but were used selectively as closely related supporting evidence. Findings from broader adult T2DM populations were similarly used only for contextual interpretation and were identified as such in the synthesis.

Methodological quality of evidence contributing to the narrative synthesis was appraised using design-appropriate tools. Primary qualitative studies were assessed using the Critical Appraisal Skills Program (CASP) Qualitative Studies Checklist, analytical cross-sectional studies using the Joanna Briggs Institute (JBI) Critical Appraisal Checklist for analytical Cross-Sectional Studies, cohort studies using the JBI critical appraisal for cohort studies and primary mixed-methods studies using the Mixed Methods Appraisal Tool (MMAT). Systematic reviews and other review-level evidence synthesis were appraised separately using the JBI Critical Appraisal Checklist for Systematic Reviews and Research Syntheses, while narrative reviews were appraised using the Scale for the Assessment of Narrative Review Articles (SANRA).

Conceptual papers, books, position statements and other background sources were not formally appraised because they were used to provide contextual or theoretical framing rather than primary evidence for the review question. Appraisal findings were used to inform the interpretation and weighting of evidence rather than as criteria for study exclusion. One reviewer completed the initial methodological appraisal, which was independently checked by a second reviewer; disagreements were resolved through discussion, with consultation from a third reviewer where required. Methodological quality was considered separately from population applicability. Studies involving young adults with youth-onset T2DM (onset<8 years) were therefore appraised according to study design but classified as closely related supporting evidence rather than direct evidence for adults diagnosed with T2DM between 18 and 39 years.

Findings were synthesised thematically across individual, relational, sociocultural and healthcare-system domains. Evidence derived directly from adults with early-onset T2DM was prioritized, while findings from young adults with youth-onset T2DM and broader adult T2DM populations were used selectively as supporting or contextual evidence and identified as such in the synthesis.

## 3 Results

The literature search yielded 2662 studies (**Supplementary Table 1**). After removing 1243 duplicates, 1419 articles remained for screening. Following review of titles and abstracts, 1293 studies were excluded based on the predefined eligibility criteria. Of 126 full texts assessed, 99 were excluded for reasons such as study population being more than 40 years old, or difficulty isolating findings from a mixed diabetes cohort. Following database screening and supplementary reference list searching, 35 sources contributed to the narrative review. These comprised 29 primary studies (12 qualitative studies, 15 cross-sectional studies, one cohort study and one mixed-methods study) and 6 review-level sources used as supporting or contextual evidence. The review-level sources included systematic reviews, one mixed-methods systematic review, one narrative review and one rapid evidence review. Studies were conducted predominantly in East Asia and the Pacific, Europe and North America (summarized in **Table 1** and **Supplementary Table S2**). To facilitate appraisal of the quantitative evidence, **Table 1** summarizes selected quantitative findings from observational studies relevant to the major themes identified in this review.

**Table 1:** Selected quantitative findings relevant to psychosocial, self-management and healthcare outcomes in young adults with early-onset T2DM.

Study	Population/design	Domain	Main quantitative finding	Interpretation/key limitation
Hessler DM et al., 2011 (10)	Community-based study of 506 adults with T2DM	Distress, self-efficacy and self-management	Younger age was associated with greater diabetes distress (β = −0.21, *p* < 0.001), lower diabetes self-efficacy (β = 0.18, *p* < 0.001), poorer diet (β = 0.20, *p* < 0.001) and higher HbA1c. Stress and diabetes distress were associated with higher HbA1c in younger but not older adults.	Supports age-related differences in psychosocial burden but represents broader adult T2DM evidence rather than a cohort defined specifically by early-onset T2DM.
Ke et al., 2019 (12)	Population-based cohort of people with young-onset T2DM	Mental-health hospitalisation	Among those aged 20–39 years, mental illness accounted for 28% of hospitalisations, corresponding to 587.2 bed-days per 1,000 patient-years. Overall, 36.8% of bed-days before age 40 were attributable to mental illness; 55.1% were attributed to psychotic disorders and 31.4% to mood disorders.	Provides population-level evidence of substantial mental-health burden in young-onset T2DM; hospital utilisation does not directly measure lived psychological experience.
Wong et al., 2024 (13)	Cross-sectional comparison of young-/early-onset and usual-onset T2DM	Self-efficacy	Young adults with early-onset T2DM had similar diabetes knowledge but significantly lower self-efficacy than those with usual-onset T2DM (adjusted B = −0.19, 95% CI −0.35 to −0.03).	Suggests that self-management difficulties may relate to confidence rather than knowledge alone; cross-sectional design precludes causal inference.
Trief et al., 2022 (11)	iCount study of emerging adults with youth-onset T2DM; mean age approximately 26 years	Medication adherence	69.8% of participants prescribed oral glucose-lowering medication and 37.0% of those prescribed insulins took <80% of prescribed treatment. Lower adherence was associated with negative medication beliefs, lower self-efficacy, diabetes distress, food insecurity and unmet material needs.	Demonstrates substantial treatment-adherence difficulty and interconnected psychosocial/material barriers; population had youth-onset rather than exclusively adult-onset T2DM.
Misra et al., 2023 (14)	National audit/population data from England	Socioeconomic disadvantage	Among younger adults, 35% of those aged 19–39 years were from the most deprived quintiles compared with 29% of those aged 40–59 years. Greater deprivation was associated with higher HbA1c, albuminuria and predicted cardiovascular risk.	Highlights socioeconomic patterning of risk; observational data cannot establish that deprivation caused poorer outcomes.
Wijayaratna et al., 2021 (15)	Cross-sectional study of young people aged 15–30 years with diabetes in Auckland	Medication access	Only 63% of young people with T2DM had diabetes medication dispensed; nearly one-third of those with severe hyperglycaemia had received no diabetes medication during the preceding 6 months.	Suggests an important treatment-access/treatment-delivery gap; age range includes participants <18 years and study included both T1DM and T2DM.
Malik et al., 2023 (16)	SEARCH for Diabetes in Youth Study	Food insecurity	Household food insecurity was reported in 30.7% of participants with T2DM compared with 17.7% of those with T1DM. Lower income, lower parental education and lack of medical insurance were independently associated with greater odds of food insecurity.	Demonstrates substantial material hardship among young people with T2DM; includes youth-onset disease and therefore is not restricted to diagnosis at 18–39 years.
Reid et al., 2022 (17)	Youth and young adults with youth-onset T2DM	Food insecurity/DKA	Household food insecurity was associated with a 3.08-fold higher odds of diabetic ketoacidosis compared with food security.	Links material hardship with a clinically important acute outcome, although observational design prevents causal inference.
Gucciardi et al., 2009 (18)	Population-based cross-sectional Canadian study	Food insecurity	Each 1-year younger age at T2DM diagnosis was associated with a 4% increase in the odds of household food insecurity (OR 1.04, 95% CI 1.02–1.05).	Association was modest and may be confounded by diabetes duration and socioeconomic disadvantage; not restricted to the review’s 18–39-year population.

This table summarizes quantitative studies only. Qualitative and mixed-methods findings are synthesized narratively in Sections 3.1–3.7 are not included in this table.

### 3.1 Young adulthood as a developmental context for early-onset type 2 diabetes

Young adulthood is a distinct developmental period characterized by identity exploration, increasing autonomy and transition into employment, relationships and family formation (9, 19). These transitions create both uncertainties and opportunities, as young adults move from the relative dependency of adolescence towards more independent and self-directed lives in work, relationships and worldviews (20). Although the concept of emerging adulthood is commonly described as spanning ages 18 to 29 years (21), adult role transitions may extend from as early as 18 to as late as 40 years, depending on social, cultural, and economic contexts (22). A diagnosis of T2DM during young adulthood may affect illness perceptions and engagement with care while routines, responsibilities and self-management behaviors are still developing. Evidence from broader adult T2DM populations suggests that a younger age is associated with greater diabetes distress, lower self-efficacy and poorer self-management outcomes (10). These challenges are not unique to young adults but they may have greater impact during young adulthood, when individuals are still developing health behaviors and self-management routines while navigating competing life demands (23). In this context, a diagnosis of early-onset type 2 diabetes may exert a lasting influence on how young adults perceive their illness, engage with care and establish self-management patterns over time.

### 3.2 Understanding diagnosis and illness meaning in early-onset type 2 diabetes

Qualitative studies suggest that young adults with early-onset T2DM may interpret their diagnosis in relation to its perceived implications for future health, relationships and family life (24). In a qualitative study conducted in individuals at 3 years following diagnosis of diabetes, Kneck et al. described living with diabetes as a continuous process of balancing, adaptation and interpretation of bodily symptoms in relation to diabetes. Uncertainty about bodily needs increased dependence on others for guidance, making diabetes self-management difficult. Participants often described diabetes as a condition that could never be fully mastered (25). Perceptions of future risk may also influence engagement with preventive care. Lake et al., in a review examining retinal-screening uptake among young adults with T2DM, identified low perceived susceptibility to diabetic retinopathy, particularly in the absence of symptoms, as a potential barrier to screening (26). This suggests that complications perceived as distant or not immediately apparent may be given lower priority during young adulthood (26). Family experience also shaped the meaning of diabetes. In a qualitative focus-group study of young people with T2DM, witnessing diabetes-related complications in relatives increased perceived seriousness and motivated self-care, whereas for others, familiarity with diabetes normalized the condition, fostering a sense of fatalism (27). Parenthood could sometimes motivate healthier behaviors while caregiving duties reduced time, energy, and attention available for self-management (27). Notably, these findings suggest that perceived immediacy of risk, personal experience of symptoms and exposure to diabetes within the family may shape how young adults understand the seriousness and relevance of their condition, although the evidence is predominantly qualitative and exploratory.

### 3.3 Identity, stigma and emotional burden in early-onset type 2 diabetes

Identity is an important dimension of living with diabetes. Diabetes identity may range from acceptance and integration to rejection and feeling overwhelmed by the condition (28). More adaptive integration has been associated with better psychosocial functioning and self-management (29), whereas negative identity and stigma were linked to concealment, avoidance, stigma, diabetes-related distress and impaired self-care (28, 30, 31). However, much of this evidence derives from mixed diabetes populations, rather than specifically from adults with early-onset T2DM. In young adults with T2DM, stigma is also closely intertwined with obesity-related stigma and weight bias, as higher body mass index is frequently associated with early onset disease. Thus, experiences of weight-related and diabetes-related stigma may compound one another and are difficult to disentangle in this population (32).

Direct qualitative evidence from young adults with T2DM suggests that stigma can influence everyday self-management. Wong et al. reported stigma and self-blame among adults aged 22 to 39 years, with some participants concealing their diagnosis or avoiding behaviors that might reveal their condition in social settings (33). Similarly, Sim et al. described stigma arising from the self, others and healthcare professionals, which could discourage treatment-related behaviors such as medication taking (24). In terms of emotional burden, population-level data also indicate a substantial mental-health burden in early-onset T2DM. In a Hong Kong population-based cohort, 36.8% of hospital bed-days before age 40 years were attributable to mental illness; among individuals aged 20–39 years, mental-health issues accounted for 28% of hospitalizations (12). Although stigma was consistently associated with concealment, distress and poorer self-management, most evidence was cross-sectional or qualitative, limiting conclusions regarding directionality. Evidence directly specific to adults diagnosed with T2DM between 18 and 39 years old was also limited.

Closely related evidence from adolescents and young adults with youth-onset diabetes is broadly consistent. In the SEARCH for Diabetes in Youth and young adults study, 22% of the participants had T2DM (mean age of 24.6 years) and within this subgroup, higher diabetes stigma scores were associated with insulin use and retinopathy (34). Broader contextual evidence from mixed diabetes populations is also consistent. In the Moderators of the Impact of Diabetes Stigma (MILES)-2 study, in which the study consists of 54% people with T2DM, diabetes stigma was associated with poorer psychological wellbeing, including greater depressive symptoms, anxiety and diabetes stress while self-esteem and social support partly mitigate its impact (35). Overall, available evidence suggests that stigma and negative diabetes identity may contribute to distress, concealment and reduced engagement with self-care. However, most studies are cross-sectional or qualitative, and the direction of these relationships remains uncertain.

### 3.4 Self-management amid competing life-stage demands

Self-management in early-onset T2DM appears to be shaped not only by diabetes knowledge, but also by confidence, emotional responses and practical demands of young adulthood. Compared with adults with later-onset T2DM, young adults with early-onset T2DM reported similar diabetes knowledge but lower self-efficacy (adjusted *B* = -0.19, CI -0.35 to -0.03), suggesting reduced confidence and capability than to knowledge deficits alone (13).

These challenges extend across different components in diabetes self-care. Direct evidence on medication adherence among young adults with T2DM remains limited, although closely related evidence from the iCount study of emerging adults with youth-onset T2DM suggests substantial difficulties with treatment adherence. At a mean age of 26 years, 69.8% of participants prescribed oral glucose-lowering medication and 37.0% of those prescribed insulins took less than 80% of their prescribed treatment. Lower adherence was associated with negative medication beliefs, lower diabetes self-efficacy, greater diabetes distress, food insecurity and unmet material needs (11). These findings suggest that adherence in young adulthood may be influenced by interacting psychological and material barriers, although their applicability to young adults first diagnosed with T2DM after 18 years should be interpreted cautiously.

Similar challenges are evident in other self-management behaviors. In a cross-sectional study of 227 adults diagnosed with T2DM at ages of 18 to 44 years old, self-care was lowest in diabetes foot care and blood glucose testing, and highest in medication taking (36). Dietary control and blood glucose testing also declined with longer diabetes duration. Qualitative evidence suggests that work and family responsibilities, weight-related concerns and diabetes stigma may limit physical activity, whereas social support, shared physical activity, and self-monitoring facilitate engagement on self-care (37). Caregiving responsibilities may be particularly complex. Britton et al. described how caregiving could motivate women with T2DM to remain healthy for their children and families, while simultaneously competing with diabetes self-management for time, energy and financial resources (38). Overall, the evidence highlights potentially bidirectional influence of caregiving, which may both support and impede self-care during young adulthood.

Importantly, these barriers do not affect all young adults in the same way. Wong et al. identified four broad patterns of self-care profiles: avoidant, indifferent, striving, and activated. The lowest level of self-care behaviors were observed in individuals with avoidant, negative emotions and indifferent mindset (33). This heterogeneity is also supported by mixed-methods evidence from Bower et al., who identified distinct perspectives among adults aged 18–40 years with T2DM, including individuals who were stressed and coping with crises, financially disadvantaged and poorly supported, well-intentioned but struggling to succeed, withdrawn and worried, or particularly affected by the stigma of having diabetes at a young age (as described in previous section) (39). Thus, these findings suggest that self-management difficulties arise from differing combinations of psychological, social and material circumstances rather than from a single uniform set of barriers. However, most available evidence was cross-sectional or qualitative and the direction of associations between distress, self-efficacy, material hardship and self-management cannot be established. These findings should therefore be interpreted as identifying potentially important barriers and patterns of need rather than demonstrating causal pathways.

### 3.5 Socioeconomic disadvantages and material barriers to self-care

Socioeconomic disadvantages may restrict access to medication, healthcare and appropriate food. In a population study in United Kingdom, younger adults were disproportionately represented in the most deprived quintiles, and deprivation was associated with poorer glycemic and cardiovascular risk profiles (14). Supporting evidence from a broader young-person cohort in Auckland, which included participants diagnosed between 15 and 30 years of age, found that only 63% of those with T2DM had diabetes medication dispensed and that nearly one-third of those with severe hyperglycemia had received no diabetes medication during the preceding six months (15). As this cohort included participants diagnosed before 18 years of age, these findings should be considered closely related rather than direct evidence for adult-onset early T2DM.

Household food insecurity (HFI) illustrates how material hardship may compromise feasibility of diabetes self-management. Direct evidence specifically examining HFI among adults diagnosed with T2DM during young adulthood is limited. However, closely related evidence is available from young adults with youth-onset T2DM. In the SEARCH study, HFI was more prevalent among participants with T2DM (30.7%) than those with T1DM (17.7%) (16). Lower income, lower parental education and lack of medical insurance were also independently associated with greater odds of HFI (16). In a subsequent analysis of young people with youth-onset T2DM, HFI had a 3.08-fold higher odds of experiencing diabetic ketoacidosis compared with those without HFI (17).

Evidence from broader adult populations provides additional context. In a Canada population-based cross sectional study, younger age at T2DM diagnosis was associated with a modest increase in the odds of HFI for each one-year decrease in age of diagnosis of T2DM (1.04 [95% CI 1.02-1.05]) (18). Among young adults in the general population, HFI was associated with poorer self-reported health status (2.63, 95% confidence interval (CI) 1.63-4.24) and diabetes (1.67, 95% CI 1.18-2.37) (40), while among working adults with diabetes, HFI was associated with more than twice the number of missed workdays (41). These studies are not specific to early-onset T2DM but support the broader conclusion that access to food, medication and healthcare may shape the practical feasibility of sustained self-management.

### 3.6 Reproductive, peers and the broader relational and sociocultural context

While section 3.2 considered how prior family experiences shape the meaning attached to a diabetes diagnosis, this section examines how reproductive expectations, peer relationships and cultural norms influence day-to-day diabetes management.

Reproductive planning represents a particularly salient concern during young adulthood. Young women with T2DM have described anxiety surrounding fertility, pregnancy-related risks and future health of their children. However, these concerns are often not discussed with healthcare professionals, leaving women to manage uncertainty without adequate support (30). A systematic review similarly identified tension between personal reproductive goals and the need to optimize diabetes management before conception (42). During pregnancy, intensified monitoring, greater treatment demands and uncertainty about fetal and maternal outcomes further increase the burden of diabetes management and undermine self-efficacy (43).

Peer relationships can have both supportive and detrimental effects on diabetes self-management. On one hand, young people with early-onset T2DM express concerns about judgment and social acceptance whereas supportive peers may facilitate healthier behaviors through shared exercise, improved dietary choices, practical assistance during emergencies, and emotional support (33). Evidence from general adult diabetes populations suggest that peer support interventions were associated with a small but statistically significant reduction in HbA1c of 0.24% (95% CI, 0.05–0.43) (44).

Sociocultural expectations influence whether self-management behaviors are acceptable and sustainable. In East Asian adults with T2DM, dietary choices were shaped not only by knowledge of healthy eating, but also by beliefs about food, family roles and the importance of maintaining social harmony (45). Participants described how following a separate diabetes-related diet could reduce enjoyment of shared meals, create tension within families or make them feel burdensome to others (45). Gendered expectations may restrict physical activity (46) or intensify stigma in some communities, particularly where diabetes or body weight affects perceptions of social or marital suitability (47). Although these studies were not specific to early-onset T2DM, they provide concrete examples of how dietary and physical-activity recommendations are negotiated within family, social and cultural contexts rather than implemented through individual motivation alone. Evidence directly examining these influences in young adults with early-onset T2DM remains limited.

### 3.7 Experiences with healthcare and preferences for support and education

Healthcare services are often perceived as insufficiently aligned with the life stage and priorities of young adults with early-onset T2DM. In a qualitative study of young adults aged 25–45 years with T2DM, participants described existing education and support programs as oriented towards older adults and insufficiently responsive to younger adults’ concerns (48). Young adults wanted practical, usable, consistent and age-appropriate advice and greater involvement in decisions about self-management (48, 49). Important life-stage issues, including pregnancy and surgery, were not always addressed at diabetes self-management education (DMSE) (48). Similarly, young women with T2DM have also described care across the pre-pregnancy, pregnancy and postpartum periods as fragmented, with unclear pathways to specialist support and disjointed transitions back to routine diabetes care (42).

Communication and service accessibility also influenced engagement (50). Participants described limited consultation time as restricting opportunities to discuss important concerns, while medical jargon could reduce understanding and discouraged help-seeking (24). They valued practical information on diet, fertility, age-related complications and medication effects, especially when delivered in a credible, individualized, empathetic and non-stigmatizing manner (24, 33, 50). Flexible appointments, remote consultations and support outside conventional working hours were viewed as more compatible with employment and caregiving responsibilities (24).

Digital platforms may offer an alternative way to improve access to diabetes education, but convenience may not necessarily ensure relevance or credibility (48). Navigating large volumes of online content, filtering conflicting messages, and judging the reliability of sources could be time-consuming and stressful. Participants preferred centralized, credible resources, and broader digital-intervention literature supports features such as personalised feedback, goal-setting and interaction (48, 51), rather than simply transferring existing educational materials online (52). These findings suggest that diabetes education for young adults with early-onset T2DM should be credible, curated and professionally supported, while also addressing diabetes distress and stigma relevant to the individual’s age and life stage (8).

Evidence on peer support was similarly mixed. Some young adults valued contact with others of a similar age as it reduced isolation and provided practical and emotional support (24) and motivated healthy behaviors such as exercise (36). Others found existing support groups poorly tailored to their age and life stage, particularly when groups are dominated by older adults or when the advice offered feels impractical given the demands of work or caregiving (36). Digitally mediated groups offer greater flexibility, but their quality depends on appropriate moderation and access to professional input (36). As an example, participation in WeChat diabetes-related groups, was positively associated with exercise management, better discussions around treatment experiences, and dietary choices (36). These findings suggest that support should be age-relevant, credible and adaptable, rather than assuming that clinic-based, digital or peer-led delivery will suit all young adults. In summary, the available evidence consistently identifies a perceived mismatch between conventional diabetes services and the priorities of younger adults. However, most findings reflect patient preferences and qualitative experiences rather than comparative evidence that models improve clinical outcomes.

## 4 Discussion

Early-onset T2DM is experienced at the intersection of substantial biological risk and developmental demands of young adulthood. Young adults reported difficulties integrating diabetes into their identities. Stigma and concealment, emotional distress, competing work and family responsibilities, financial hardships, and healthcare services were perceived as insufficiently aligned with their priorities. Although many of these challenges also occur in later-onset T2DM, they may be particularly consequential during a period characterized by increasing autonomy, unstable routines, family formation, reproductive planning and limited financial flexibility. The evidence varied considerably, with the most consistent findings relating to stigma, diabetes distress, reduced self-efficacy and the perceived mismatch between standard services and young adults’ needs. However, much of the evidence was cross-sectional or derived from small qualitative studies. Findings should therefore be interpreted as identifying important care priorities rather than demonstrating that these experiences are unique to early-onset T2DM or that particular service models improve clinical outcomes.

Clinicians should also consider the differential diagnosis of early-onset diabetes. A minority of individuals diagnosed with apparent T2DM before 40 years of age may instead have maturity-onset diabetes of the young (MODY) or other forms of monogenic form of diabetes. These individuals may have strong family history of diabetes, atypical body habitus or preserved beta-cell function and often require distinct treatment or prognostic family-screening approaches (53). Validated MODY probability calculators are available to support clinician in identifying individuals for genetic testing. However, these calculators were derived and validated predominantly in European cohorts, and their diagnostic accuracy in Asian populations remains unclear, with some studies reporting reduced performance (54, 55). Population-specific diagnostic algorithms incorporating clinical and biochemical features have been developed to better identify candidates for genetic screening among Asian patients with early-onset T2DM and more studies are needed to validate this (5).

Developmentally appropriate care should extend beyond the provision of diabetes knowledge to support confidence and capability for sustained self-management (**Figure 1**). Qualitative evidence consistently favors flexible access, age-appropriate communication, collaborative goal setting, routine assessment of diabetes stigma and distress such as using validated instruments like Problems Areas in Diabetes questionnaire, which has been used to assess diabetes-related distress in both type 1 (56) and type 2 diabetes populations (57), or Diabetes Distress Scale (DDS) (58) and counselling for reproductive issues where relevant. Issues such as food insecurity, medication affordability and workplace constraints should be considered where appropriate. Self-efficacy may be an important component of sustainable diabetes self-management, given its relationship with self-care behaviors, glycaemic outcomes and quality of life in the broader T2DM population. However, whether interventions specifically targeting self-efficacy improve these outcomes in young adults with early-onset T2DM remains uncertain and requires prospective evaluation.

**Figure 1:**
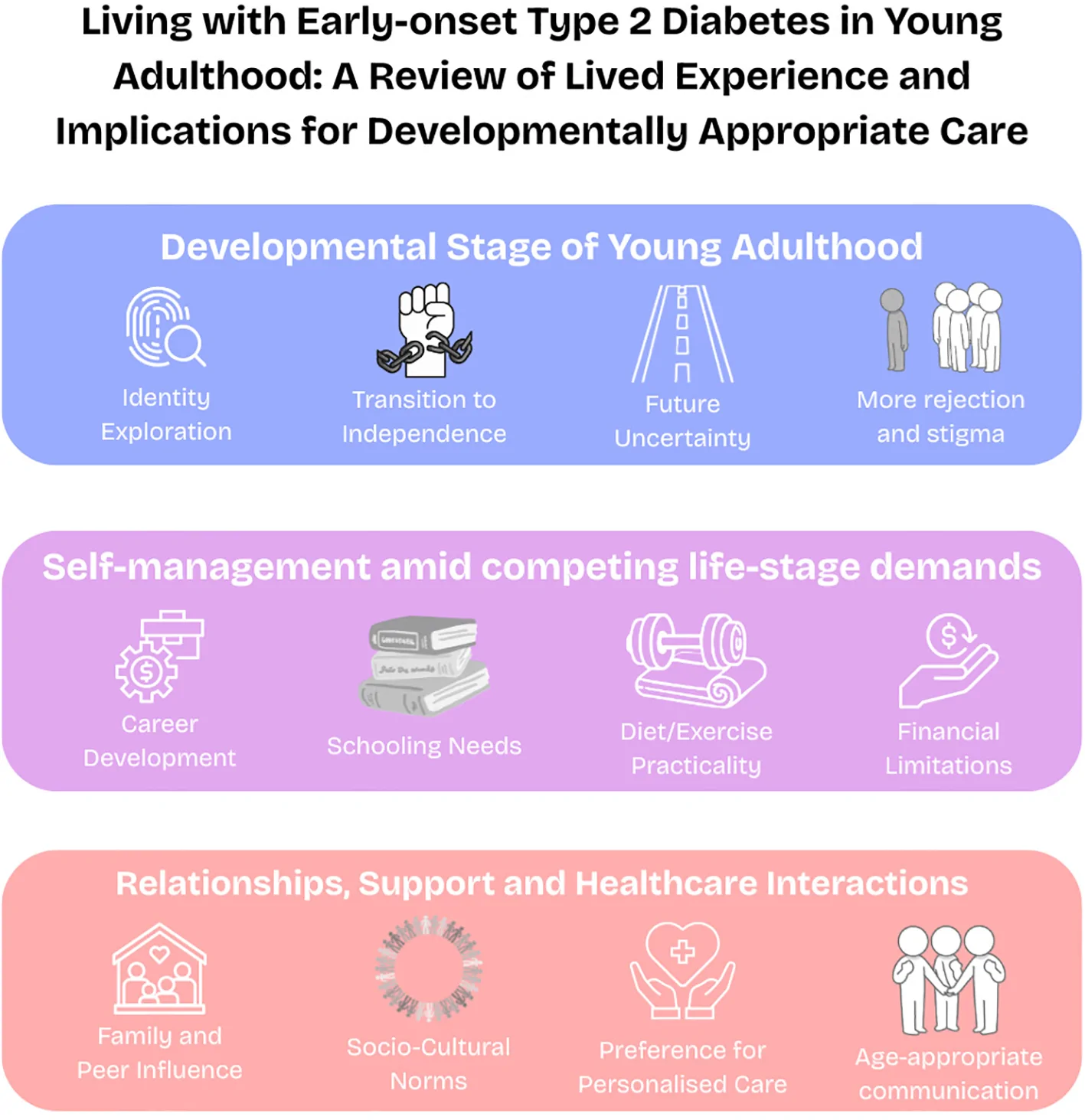
The image shows the various factors that highlight the perspectives of individuals with early onset T2DM. Image created via graphic program: “Canva”.

Young adults valued credible, curated digital education; and opportunities for age-relevant peer support. These approaches should ideally be co-designed with patients, because acceptability alone does not establish effectiveness. Their clinical impact should be evaluated using patient-reported outcomes, glycaemic and cardiometabolic endpoints, engagement, healthcare utilisation and equity outcomes. Evidence from the broader adult T2DM literature supports interrelationships between self-efficacy, self-care behaviors, glycaemic control and quality of life, although age-specific pathways in early-onset T2DM remain insufficiently characterized.

At the health-system level, early-onset T2DM should be identifiable in dedicated audit categories, service evaluation and outcome monitoring. Priorities include improving affordability of medications and monitoring technologies, strengthening reimbursement and infrastructure for hybrid care, and promoting workplace policies that support diabetes care without stigma or penalty. There is also a need to recognize the broader societal and intergenerational implications of early-onset T2DM and including productivity loss, reproductive risk, intergenerational transmission of diabetes risk.

Future research should test co-designed models of care. Priorities include determining which service components improve engagement and outcomes; evaluating interventions to reduce stigma, concealment and diabetes distress; testing digital and hybrid models over meaningful follow-up periods; and quantifying workplace, educational and socioeconomic barriers.

Several limitations should be acknowledged. The narrative design and English-language restriction may have introduced selection bias. Definitions of early-onset T2DM and young adulthood varied, and some studies used upper age thresholds beyond 39 years or included mixed diabetes populations. Where direct evidence from adults diagnosed with T2DM between 18 and 39 years was limited, we selectively used findings from young adults with youth-onset T2DM as closely related supporting evidence; these findings should not be assumed to be fully generalisable to adult-onset early T2DM. Much of the direct evidence was cross-sectional or derived from small, single-site qualitative samples, limiting causal inference and transferability. Broader adult T2DM studies were used selectively as contextual evidence and should not be interpreted as demonstrating age-specific effects. The literature was geographically concentrated in East Asia and the Pacific, Europe and North America, with limited evidence from the Middle East and North Africa, South Asia, Latin America and many low- and middle-income countries. As stigma, family roles, employment, food practices, healthcare access and financial hardship are socioculturally contingent, generalisability to underrepresented settings is uncertain.

## 5 Conclusion

Early-onset T2DM occurs during a life stage marked by changing identity, autonomy, employment, relationships and family planning. These circumstances may intensify diabetes distress, stigma, self-management difficulties and disengagement from health services. **Table 2** summarizes how these barriers to care may arise, their consequences and how developmentally appropriate care could address them. Care should combine proactive risk-factor management with flexible, collaborative and non-stigmatizing support. Co-designed intervention studies are needed to determine whether developmentally appropriate models improve patient-reported, clinical and health-service outcomes.

**Table 2:** Showing the potential gaps in current care, associated unmet needs and priorities for developmentally appropriate care in early-onset type 2 diabetes.

Reasons for inaction	Consequences of inaction	What developmentally appropriate care could address
• Existing adult diabetes pathways may be assumed to be sufficient • Younger adults may be perceived as having lower immediate complication risks • Absence of overt complications may reduce perceived urgency • Conventional service models may not accommodate work, caregiving and family responsibilities • Poor attendance may be interpreted as disengagement rather than reflecting practical barriers • Early-onset T2DM may not be routinely identified as a distinct group in audit and service evaluation	• Persistent diabetes distress, stigma, and concealment • Reduced confidence and engagement with self-management • Medication non-adherence and difficulty sustaining self-care behaviors. • Reproductive concerns and preconception needs may remain insufficiently addressed. • Material hardship, food insecurity and workplace barriers may compromise diabetes management • Psychological and healthcare needs remain underappreciated and unrecognized • Outcomes and inequalities in this population may remain poorly characterized.	• Proactive cardiometabolic risk assessment and management • Flexible access and age-appropriate, non-stigmatising communication • Collaborative goal-setting and support for self-efficacy • Integrated reproductive and preconception counselling where relevant • Assessment of social and material barriers with appropriate health-social care support • Curated digital/hybrid education and age-relevant peer support • Disaggregated audit, service evaluation and outcome monitoring.
