# Supplementary material for: Living with early-onset type 2 diabetes in young adulthood: a narrative review of lived experience and implications for developmentally appropriate care
Source: Front Endocrinol (Lausanne). 2026 Sep 22;17:1903159. doi: 10.3389/fendo.2026.1903159 (PMC13640145; doi:10.3389/fendo.2026.1903159)
Supplement: Supplementary file 1 [file SupplementaryFile1.docx]

Supplementary Table S1a: Full database search strategies. Searches were conducted from database inception to 31 August 2025 and restricted to English-language publications. Search syntax was adapted to the indexing and field conventions of each database. Reference lists of included studies and relevant reviews were screened separately for additional eligible studies.

| Database/Platform | Search Terms | Coverage | Limits | Records retrieved |
| --- | --- | --- | --- | --- |
| PubMed | ("type 2 diabetes"[Title/Abstract] OR "Diabetes Mellitus, Type 2"[MeSH Terms]) AND ("early onset"[Title/Abstract] OR "young onset"[Title/Abstract] OR "younger onset"[Title/Abstract] OR "young adult"[Title/Abstract] OR "emerging adult"[Title/Abstract]) AND (perception*[Title/Abstract] OR experience*[Title/Abstract] OR belief*[Title/Abstract] OR concern*[Title/Abstract] OR attitude*[Title/Abstract] OR stigma*[Title/Abstract] OR distress[Title/Abstract] OR psychosocial[Title/Abstract] OR self-management[Title/Abstract] OR self-care[Title/Abstract] OR adherence[Title/Abstract] OR identity[Title/Abstract] OR healthcare[Title/Abstract] OR "health care"[Title/Abstract] OR support[Title/Abstract] OR qualitative[Title/Abstract] OR interview*[Title/Abstract] OR "focus group*"[Title/Abstract]) | Inception to 31 August 2025 | English | 1333 |
| Medline via Ovid | 1. exp Diabetes Mellitus, Type 2/ OR ("type 2 diabetes" or "type 2 diabetes mellitus").  2. ("early onset" or "young onset" or "younger onset" or "young adult*" or "emerging adult*").  3. (perception* or experience* or belief* or concern* or attitude* or stigma* or distress or psychosocial or self-management or self-care or adherence or identity or healthcare or "health care" or support or qualitative or interview* or "focus group*").  4. 1 AND 2 AND 3 | Inception to 31 August 2025 | English | 810 |
| Embase via Ovid | 1. exp type 2 diabetes mellitus/ OR ("type 2 diabetes" or "type 2 diabetes mellitus").  2. ("early onset" or "young onset" or "younger onset" or "young adult*" or "emerging adult*")  3. (perception* or experience* or belief* or concern* or attitude* or stigma* or distress or psychosocial or self-management or self-care or adherence or identity or healthcare or "health care" or support or qualitative or interview* or "focus group*")  4. 1 AND 2 AND 3 | Inception to 31 August 2025 | English | 519 |

Supplementary Figure 1: Flow chart of literature search for our narrative review. References used for clinical framing or background contextual evidence are not included in this flow chart

**Supplementary Table S2A. Methodological appraisal of primary evidence contributing to the narrative synthesis.** Methodological quality was assessed using design-appropriate tools. Qualitative studies were appraised using the Critical Appraisal Skills Programme (CASP) Qualitative Studies Checklist, analytical cross-sectional studies using the Joanna Briggs Institute (JBI) Critical Appraisal Checklist for Analytical Cross-Sectional Studies, cohort studies using the JBI Critical Appraisal Checklist for Cohort Studies, and primary mixed-methods studies using the Mixed Methods Appraisal Tool (MMAT). Methodological quality was distinguished from population applicability; thus, a study could be methodologically robust while still providing indirect evidence for the population of interest. Appraisal findings were used to inform interpretation and weighting of the evidence rather than as criteria for automatic exclusion. Applicability to adults diagnosed with early-onset type 2 diabetes mellitus (T2DM) between 18 and 39 years of age was considered separately from methodological quality. Overall methodological concerns were summarised narratively as minor, moderate or major rather than converted into numerical quality scores.

Abbreviations: CASP, Critical Appraisal Skills Programme; JBI, Joanna Briggs Institute; T2DM, type 2 diabetes mellitus.

| **Study** | **Appraisal tool** | **Sampling** | **Measurement/data collection** | **Analysis/confounding** | **Follow-up/reflexivity** | **Overall methodological concerns** |
| --- | --- | --- | --- | --- | --- | --- |
| Hessler DM *et* *al.* 2011 [11] | JBI analytical cross-sectional | Community/clinical adult T2DM sample aged 20 to 80 years old (n=506); broader adult population rather than a cohort defined by early-onset T2DM. | Validated psychosocial and self-management measures; HbA1c included. | Multivariable analyses assessed age-related associations; residual confounding and age/age-at-diagnosis distinctions require care. | No follow-up. | Moderate concerns: analytically useful contextual evidence, but indirect to the target population and cross-sectional. |
| Trief PM *et al.* 2022 [12] | JBI analytical cross-sectional | Cohort of young adults with youth-onset T2DM recruited from 2014 to 2020 in the follow up phase of TODAY study (n=572, mean age is 26), relevance to youth-onset T2DM < 18 years old | Medication adherence measured using unannounced pill counts for oral agents and self-report for insulin; psychosocial measures validated. | Multivariable associations assessed; measurement differences between oral and insulin adherence and cross-sectional timing limit inference. | The cited 2022 analysis is cross-sectional; a separate 2023 longitudinal iCount paper provides 1-year predictors and should be cited separately if used. | Moderate concerns: objective pill counts are a strength, but youth-onset indirectness, mixed adherence measurement and cross-sectional analysis. |
| Sim RRJ *et* *al.* 2024 [18] | CASP qualitative | Maximum-variation sampling across age, ethnicity and marital status; 21 participants aged 22 to 39 from three Singapore polyclinics. Small but purposively diverse sample. | In-depth semi-structured interviews appropriate to the question; thematic coding/conceptualisation used. | Analysis aligned with aims and produced clearly supported themes; full-text confirmation of independent coding/audit trail should be retained in reviewer worksheet. | Researcher-participant relationship described; no longitudinal follow-up expected. | Minor concerns: strong design-question fit and purposive variation, with limits mainly from small single-health-system sample and reflexivity reporting. |
| Kneck A *et* *al*. 2012 [19] | CASP qualitative | 13 participants diagnosed with diabetes 3 years previously aged 20 to 80 years old; Swedish study, small sample and diabetes type/age specificity are not aligned closely with the review population. | Narrative interviews using a lifeworld approach; phenomenological-hermeneutic analysis appropriate to experiential question. | Interpretive analysis is congruent with methodology, but small sample and population indirectness constrain transferability. | Researcher- participant relationship not fully described | Moderate concerns: qualitative credibility may be acceptable, but major applicability/indirectness to early-onset adult T2DM. |
| Gopalan A *et* *al*. 2022 [21] | CASP qualitative | 41 adults aged 21–44, diagnosed within prior 2 years, from an integrated California health system; purposive racial/ethnic/geographic recruitment. Participation/nonresponse may select more engaged individuals. | Six focus groups, each meeting twice; transcripts thematically coded and mapped to COM-B. Focus groups well suited to eliciting shared and contrasting family experiences. | Structured thematic approach; sample adequacy was considered a priori with option for additional groups. Full coding-team/reflexivity details should be retained in worksheet. | Repeated group meetings add depth; researcher-participant relationship described. | Minor concerns: strong relevance and transparent recruitment, but health-system/English-language selection and focus-group participation may limit transferability. |
| Bień A *et* *al.* 2016 [23] | JBI cross-sectional | Pregnant women with diabetes, not restricted to early-onset T2DM but provides pregnancy context concerns | Questionnaire measures of quality of life/illness acceptance | Associated analysis: confounding and diabetes type specificity varies | N/A | Moderate, relevant to illness acceptance; indirect population and cross-sectional design |
| Celik A *et* *al*. 2023 [24] | CASP qualitative | 36 women aged 20–45 from UK and Turkey recruited via social media, charities, employers and women's organisations; purposive online recruitment may favour more engaged participants. | 28 video interviews audio-recorded/transcribed verbatim plus 8 email interviews; Framework Analysis underpinned by self-efficacy theory; translation/transcription checks reported. | Framework Analysis appropriate and transparent; multiple data modes may produce different depth of data but allow inclusion of diverse participants. | Researcher relationship with participant not described; cross-country design strengthens breadth but cultural differences require interpretation. | Minor concerns: rich, directly relevant data and transparent collection; selection bias, mixed interview modes and transferability remain. |
| Schmitt A *et* *al*. 2014 [25] | JBI analytical cross-sectional | Mixed diabetes clinical sample (n=320, 96 were T2DM) not specific to young-onset T2DM, used as a broader context | Diabetes acceptance, self-care and glycaemic control measured with established instruments/clinical measures. | Associational analysis cannot establish whether acceptance drives self-care or reflects disease experience; confounding strategy should be confirmed from full text. | No follow-up. | Moderate concerns: useful contextual association, but cross-sectional and population indirectness. |
| Wong SKW *et* *al*. 2024 [27] | CASP qualitative | Maximum-variation sample of 21 adults aged 22-39 from Singapore polyclinics; deliberately varied age, ethnicity, education and marital status. | In-depth semi-structured interviews; thematic analysis mapped barriers/facilitators to the Theoretical Domains Framework. | Theory-informed analysis appropriate; four self-care mindset patterns generated from a small sample should be treated as interpretive typologies, not prevalence categories. | Researcher relationship with participant not described | Minor concerns: strong relevance and method fit; small single-system sample and interpretive typology limit transferability. |
| Ke C *et* *al*. 2019 [28] | JBI cohort | Large population-based young-onset T2DM cohort with comparison by age of onset; strong coverage from routinely collected health data (36.8% early-onset T2DM) | Hospitalisation and diagnostic outcomes derived from administrative/clinical records; objective but dependent on coding accuracy and healthcare-contact patterns. | Cohort analyses allow temporal outcome assessment and adjustment, though unmeasured confounding and misclassification remain possible. | Longitudinal follow-up is a major strength; adequacy/completeness of follow-up should be documented in reviewer worksheet. | Minor to moderate concerns: strong population-based longitudinal design; residual confounding and routine-data misclassification are principal limitations. |
| Eitel KB *et* *al*. 2023 [29] | JBI analytical cross-sectional | Multicentre SEARCH youth/young-adult cohort (n=1608) Mixed diabetes group consisting of 353 (22%) youth-onset T2DM. | Stigma scale plus clinical variables/outcomes; objective clinical data strengthen outcome measurement. | Adjusted analyses can identify association but not temporal direction; subgroup estimates may be imprecise. | No temporal inference from cross-sectional stigma assessment. | Moderate concerns: multicentre and clinically measured outcomes are strengths; cross-sectional design, mixed diabetes population and subgroup size limit inference. |
| Holmes-Truscott E *et* *al.* 2020 [30] | JBI analytical cross-sectional | Large national Australian diabetes survey (n=2088), mixed diabetes types and broader age distribution; 54% T2DM, used as contextual rather than direct evidence. | Validated psychosocial measures; some outcomes, including glycaemic indicators, may be self-reported depending on analysis. | Adjusted moderation/regression analyses; residual confounding and cross-sectional directionality remain. | No follow-up. | Moderate concerns: robust psychosocial measurement, but population indirectness and cross-sectional design. |
| Wong SKW *et* *al*. 2024 [31] | JBI analytical cross-sectional | 97 early-onset and 312 usual-onset T2DM participants; clinically meaningful comparator but healthcare-based recruitment may select engaged patients. | Diabetes knowledge, self-efficacy and barrier measures were structured/validated. | Adjusted comparisons accounted for multiple demographic/clinical variables; residual confounding remains. | No follow-up. | Minor concerns: well-defined comparator and adjusted analysis; cross-sectional design prevents causal inference. |
| He Q *et* *al*. 2023 [32] | JBI analytical cross-sectional | 227 young adults with early-onset T2DM (18 to 44 years old) recruited from two Chinese hospitals; clinical sampling may not represent community/non-attending patients. | Self-management and related psychosocial factors assessed using questionnaires/standardised measures. | Regression modelling used to examine related factors; cross-sectional design and model-selection choices may permit residual confounding. | No follow-up. | Moderate concerns: directly relevant sample and structured measurement, but two-site clinical sampling and cross-sectional design. |
| Zhao X *et* *al*. 2025 [33] | CASP qualitative | Purposive sampling of adults aged 18 to 40 with young-onset T2DM, designed for variation in relevant characteristics; recruitment route should be considered for self-selection. | Semi-structured interviews; Framework Analysis/theory-informed COM-B/TDF approach appropriate to physical-activity barriers/facilitators. | Transparent framework-based coding; credibility strengthened by theoretical mapping, but findings are context-specific and not prevalence estimates. | Researcher reflexivity not described and sampling saturation documented | Minor concerns: strong directness and method fit; self-selection and transferability are main concerns. |
| Britton LE *et* *al*, 2023 [34] | CASP qualitative | 32 Black and or Latina women aged 18 to 40 years with T2DM | Semi-structured interviews, secondary analysis of parent study data | Content analysis appropriate for exploring caregiving and self-management | No follow up | Minor concerns, directly relevant population and appropriate qualitative methods but transferability and secondary analysis limits the study, limited by reflexivity. |
| Bower P *et* *al.* 2023 [35] | MMAT mixed methods | Adults aged 18 to 40 years with confirmed T2DM in Greater Manchester; 46 completed Q-sort and 43 included in final analysis | Demographic questionnaire, 64-item Q sort, focus groups and interviews | Q-method analysis integrated with qualitative findings to identify and interpret five perspectives | No follow up | **Minor concerns:** strong alignment of design, data and integration with the research question; main concerns relate to sampling and subgroup size, main limitations are small numbers |
| Misra S *et* *al*. 2023 [36] | JBI analytical cross-sectional | Whole-population National Diabetes Audit dataset (n=122,780); 98% aged 18 to 39 years old. | Routinely collected age, ethnicity, deprivation, HbA1c, weight and care-process data; objective but completeness/missingness may vary. | Pre-specified age-band comparisons are appropriate; confounding adjustment is limited for some descriptive comparisons. | Cross-sectional audit period; no individual temporal inference. | Minor concerns: excellent population coverage and sample size; routine-data completeness and descriptive cross-sectional design are main limitations. |
| Wijayaratna S *et* *al*. 2021 [37] | JBI analytical cross-sectional | Young people with T1DM/T2DM diagnosed aged 15 to 30 in Auckland; sizeable clinical cohort but includes minors and mixed diabetes types, used as broader evidence | Clinical risk factors and deprivation indices largely objective/routine data. | Associations between socioeconomic status and risk factors assessed; residual confounding and mixed diabetes population require caution. | No follow-up for cited analysis. | Moderate concerns: objective clinical measures and sizeable sample, but mixed diabetes types/age range and cross-sectional design limit directness. |
| Malik FS *et* *al.* 2023 [38] | JBI analytical cross-sectional | 2,561 young adults, including 384 with T2DM, from multicentre SEARCH; good geographical breadth | HFI assessed with validated 18-item US Household Food Security Survey Module; insurance/income/education captured. | Multivariable logistic regression used to identify correlates; residual socioeconomic confounding remains. | No follow-up for HFI prevalence analysis. | Minor concerns: strong validated exposure measure and multicentre sample; cross-sectional design and youth-onset indirectness. |
| Reid LA *et* *al.* 2022 [39] | JBI analytical cross-sectional | Youth/young adults with youth-onset T2DM; multicentre but not adult-onset early T2DM. | Validated food-security measure; HbA1c measured; DKA/severe hypoglycaemia based on recent-event reporting/records depending on study protocol. | Adjusted regression analyses appropriate; event rarity can yield imprecise estimates and cross-sectional exposure/outcome timing limits causal inference. | No longitudinal temporal ordering for cited analysis. | Moderate concerns: relevant clinical outcome and adjusted analysis; cross-sectional design, youth-onset indirectness and possible event-reporting imprecision. |
| Gucciardi E *et* *al*. 2009 [40] | JBI analytical cross-sectional | Large Canadian population survey; broad age range and not specific to early-onset T2DM. | Food insecurity from standard population measure; diabetes/age at diagnosis may be self-reported. | Population-weighted regression possible; residual socioeconomic confounding and disease-duration effects complicate interpretation. | No follow-up. | Moderate concerns: large population sample but substantial indirectness and cross-sectional/self-report limitations. |
| Nagata JM *et* *al.* 2019 [41] | JBI analytical cross-sectional | Nationally representative US young-adult cohort dataset; broad chronic-disease population rather than early-onset T2DM specifically. | Food insecurity and health conditions largely self-reported in survey data. | Adjusted analyses can address measured confounders but cannot establish temporality. | Source cohort is longitudinal, but cited exposure/outcome analysis is cross-sectional. | Moderate concerns: strong population sampling; diabetes-specific inference is indirect and self-report/cross-sectional limitations remain. |
| Weinstein JM *et* *al*. 2022 [41] | JBI analytical cross-sectional | Large nationally representative working-age US adults with diabetes; broad age group, not early-onset-specific. | NHIS food insecurity, missed workdays and healthcare-use measures; substantial self-report. | Survey-weighted adjusted analyses appropriate; residual confounding and healthy-worker/working-age selection possible. | No individual longitudinal follow-up. | Moderate concerns: strong national policy relevance and size, but self-report, cross-sectional design and population indirectness. |
| Tschirhart H *et* *al.* 2024 [44] | CASP qualitative | 18 pregnant women with pre-existing T1DM/T2DM sampled from quantitative strand using nested/stratified/theoretical sampling; mixed diabetes types and specialist-clinic context. | In-depth one-to-one interviews; interpretive description and constant comparative analysis appropriate. | Iterative theoretical sampling/constant comparison strengthen analytic depth; T2DM-specific findings may not always be separable. | Researcher reflexivity partially reported to some extent in full text; no longitudinal follow-up needed. | Minor concerns: rigorous qualitative approach, but small mixed-diabetes specialist sample limits directness/transferability. |
| Sidhu T *et* *al.* 2022 [47] | CASP qualitative | 20 participants were interviewed (12 South Asians, 8 Whites, 60% T2DM), median age 60.2 years, sampling provides broader T2DM context and sociocultural context | Qualitative interviews appropriate to exploring social norms and physical activity. | Thematic/interpretive analysis; transferability depends on setting and participant characteristics | Researcher and participant relationship not described | Moderate: useful sociocultural context |
| Singh H *et* *al.* 2012 [48] | CASP qualitative | 20 outpatients (12 British South Asian, 8 White) with good/poor glycaemic control from one UK hospital; purposive comparison but small single-site sample. | Semi-structured interviews analysed using a form of Interpretative Phenomenological Analysis. | Interpretive analysis appropriate to experiences; ethnic-group comparisons from very small numbers should be treated cautiously. | Reflexivity/researcher-participant relationship should be checked in full text. | Moderate concerns: appropriate qualitative approach, but very small single-site sample and subgroup comparison limit transferability. |
| Savage S *et* *al.* 2009 [49] | CASP qualitative | 13 Type 2 diabetes aged between 25 to 45 living in Australia | Qualitative interviews/focus groups appropriate to preferences for diabetes education and support. | Thematic analysis suitable; details of analytic rigour should be retained in reviewer worksheet. | Researcher is not related to participant | Minor, relevant experiential evidence; sample size/setting; Australian based study may limit transferability. |
| Rasmussen B *et* *al.* 2016 [50] | CASP qualitative | 26 participants (12 Australia, 14 Denmark), aged 19-42 with T2DM duration >10 months; cross-country sampling adds contextual breadth. | In-depth semi-structured interviews; inductive descriptive thematic/content analysis with comparative coding. | Analysis appropriate for comparing similarities/differences across settings; sample remains small and healthcare-system differences may shape findings. | Researcher-participant relationship not described | Minor concerns: strong direct relevance and cross-country perspective; small sample and contextual heterogeneity limit transferability. |

Supplementary Table 2B **Methodological appraisal of evidence contributing to the narrative synthesis.** Methodological appraisal of review-level evidence contributing to the narrative synthesis. Systematic reviews, meta-analyses and other systematic evidence syntheses were appraised using the Joanna Briggs Institute (JBI) Critical Appraisal Checklist for Systematic Reviews and Research Syntheses. Narrative reviews were appraised using the Scale for the Assessment of Narrative Review Articles (SANRA).

**Abbreviations:** JBI, Joanna Briggs Institute; T2DM, type 2 diabetes mellitus.

| **Study** | **Review type** | **Appraisal tool** | **Role in manuscript** | **Applicability/interpretive note** | **Overall methodological concerns** |
| --- | --- | --- | --- | --- | --- |
| Lake AJ *et* *al.* 2018 [20] | Narrative review | SANRA | Used as supporting evidence regarding perceived complication risk and retinal-screening engagement | Directly focused on young adults aged 18–39 years with T2DM | **Minor–moderate** **concerns**: clinically relevant and directly aligned with the target age group, but literature-search and study-selection methods are insufficiently reported for systematic-review-level reproducibility; findings therefore used as supporting rather than definitive evidence. |
| Hopkins L *et* *al.* 2025 [43] | Systematic review of qualitative studies | JBI systematic reviews/research syntheses | Supporting reproductive-care evidence | Relevant to women with T2DM across reproductive periods; not restricted to adult-onset 18–39. | **Minor-moderate:** systematic approach and qualitative synthesis support credibility, but applicability is limited by heterogeneity in diabetes type, reproductive stage and age at diagnosis. |
| Patil SJ *et* *al.* 2016 [45] | Systematic review/meta-analysis | JBI systematic reviews/research syntheses | Contextual peer-support intervention evidence | General adult diabetes population; not age-specific. | **Moderate concerns:** synthesis provides useful intervention-level evidence, but substantial heterogeneity in populations, peer-support models and outcomes limits direct inference for young adults with early-onset T2DM. |
| Li-Geng T *et* *al.* 2020 [46] | Mixed-methods systematic review | JBI systematic reviews/research syntheses | Contextual sociocultural/dietary evidence | East Asian American adults with T2DM; older/general adult population. | **Minor–moderate concerns:** mixed-methods synthesis offers broad contextual insight, although heterogeneity in study designs and populations and limited age-specific evidence reduce applicability to early-onset T2DM. |
| Ramadas *et* *al.* 2011 [52] | Systematic review | JBI systematic reviews/research syntheses | Contextual digital-intervention evidence | General adult T2DM; older evidence base and not early-onset-specific. | **Moderate concerns:** systematic review methodology provides useful contextual evidence, but the evidence base is dated, interventions are heterogeneous and findings are not specific to young adults or contemporary digital care. |
| Gabarron *et* *al.* 2018 [53] | Rapid evidence review | JBI systematic reviews/research syntheses, interpreted with rapid-review caveat | Contextual social-media/digital evidence | Heterogeneous diabetes populations/interventions; not age-specific. | **Moderate concerns:** useful overview of an emerging evidence base, but rapid-review methods, heterogeneity of populations and interventions, and limited early-onset-specific evidence restrict confidence and direct applicability. |

Supplementary Table S2C. **Conceptual, guidance and background sources used to contextualise the narrative synthesis.** This table summarises sources used to provide epidemiological, developmental, theoretical, clinical, service-delivery or communication context for the review. These sources included empirical studies, clinical trials, secondary analyses, conceptual papers, narrative reviews, books, position statements, consensus statements and guidance documents. Because these sources were not treated as primary empirical evidence for the review question, formal methodological appraisal using JBI or CASP tools was not undertaken. Their role was limited to contextualising and interpreting findings from the primary evidence and supporting the developmental and clinical framing of early-onset T2DM.

| Source | Source type | Role | Formal appraisal |
| --- | --- | --- | --- |
| Duncan BB *et* *al.* 2025 [1] | Epidemiological report | Background epidemiological context on global T2DM prevalence and projected burden | Not formally appraised, used only for epidemiological framing |
| Misra S *et* *al.* 2023 [2] | Clinical review | Clinical context on the definition, epidemiology and characteristics of early-onset T2DM | Not formally appraised as evidence in the review, used for clinical framing |
| Sattar N *et* *al.* 2019 [3] | Population based observational cohort study | Evidence on associations between younger age at T2DM diagnosis and cardiovascular/mortality risk | Not formally appraised because it was used only to establish clinical/prognostic context |
| Arslanian S *et* *al*. [4] | Clinical trial analysis | Background clinical context comparing disease burden and characteristics of early- versus later-onset T2DM | Not formally appraised because it was used only to establish clinical/prognostic context |
| Wang M *et al.* 2023 [5] | Population-based cohort study | Background clinical context comparing disease burden and characteristics of early- versus later-onset T2DM | Not formally appraised because it was used only to establish clinical/prognostic context |
| Jiang Y, 2024 [6] | Cross-sectional observational study | Background clinical context on cardiometabolic characteristics of early-onset T2DM | Not formally appraised because it did not contribute to the thematic synthesis |
| Zeitler P *et* *al.* 2024 [7] | Post hoc analysis of clinical trials | Background treatment/clinical context for early-onset T2DM | Not formally appraised because it was used only for clinical framing |
| Holman RR *et* *al.* 2008 [8] | Long-term follow-up of randomized trial | Background evidence supporting the long-term importance of glycaemic management | Not formally appraised because it was used only to establish general clinical context |
| Misra S *et* *al.* 2022 [9] | Service-development/review article | Clinical/service context | No primary-study risk-of-bias appraisal |
| Lui JNM *et* *al.* 2026 [10] | Observational empirical study | Contextual evidence on occupation and productivity burden associated with young-onset T2dM | Not formally appraised as not used as evidence in the narrative review |
| Arnett JJ, 2000 [13] | Conceptual developmental theory | Young-adulthood framework | Not applicable |
| Arnett JJ, 2014 [14] | Book/developmental framework | Young-adulthood framework | Not applicable |
| Seiffge-Krenke I, 2013 [15] | Developmental/life-course source | Timing of adult role transitions | Background; not part of lived-experience evidence appraisal |
| Society for Adolescent Health and Medicine 2017 [16] | Position statement | Young-adult age/life-stage framing | Not applicable |
| Frech A, 2012 [17] | Empirical life-course behavioural study | Developmental context | Could be appraised with cohort/longitudinal criteria if treated as included empirical evidence; in current manuscript it functions as background context |
| Commissariat PV *et* *al.* 2025 [22] | Narrative/conceptual review | Diabetes identity framework | No primary-study risk-of-bias appraisal |
| Puhl et al. 2020 [26] | Cross-sectional study to examine intersection of weight stigma with diabetes stigma. This is not included in main analysis as it is not restricted to early-onset T2DM as defined | Used as contextual data to illustrate the associational analysis linking weight stigma and diabetes stigma | Not formally appraised as this is used as a framing for association between diabetes and weight related stigma. |
| Dickinson *et* *al.* 2017 [51] | Consensus/language guidance | Non-stigmatising communication | Not applicable |
| Shields et al. 2012 [54] | UK based clinical prediction study using n=278 type 1 diabetes and n=319 type 2 diabetes and n=594 patients with MODY | Used in discussion as a contextual framing for development and validation of probability of MODY in patients with young-onse diabetes | Not formally appraised |
| Zhao J et al. 2024 [55] | Population-based study | Used in discussion as an evidence that MODY calculators need further validation in Asian population | Not formally appraised as this is used as part of discussion to illustrate the importance of developmentally appropriate care |
| Alarcon G et al, 2025 [56] | Retrosepective cohort study in Hispanic youth with diabetes in a large paediatric centre in United States | Used in discussion as an evidence that MODY calculators may overestimate MODY risk in non-White population | Not formally appraised as this is used as part of discussion to illustrate the importance of developmentally appropriate care |
| Tan CSH et al. 2025 [57] | Singapore-based cohort study | Used in discussion as an evidence that MODY calculators may not always be applicable in Asian population | Not formally appraised as this is used as part of discussion to illustrate the importance of developmentally appropriate care |
| Grulovic et al. 2022 [58] | Cross-sectional study to evaluate diabetes-related distress in adults with type 1 diabetes | Used for discussion to validate the use of problem areas in diabetes (PAID) questionnaire in development of care | Not formally appraised as this is used as a framing for developmentally appropriate care for this group of patients. |
| Papathanasiou A et al. 2012 [59] | Cross-sectional psychometric evaluation survey assessing use of Problem Areas in Diabetes scale in individuals with type 2 diabetes | Used for discussion to validate the use of problem areas in diabetes (PAID) questionnaire in development of care | Not formally appraised as this is used as a framing for developmentally appropriate care for this group of patients. |
| Polonsky WH et al. 2005 [60] | Cross-sectional study design to assess the 17-item Diabetes Distress Scale to measure diabetes specific emotional burden in individuals with type 2 diabetes | Used for discussion to validate the use of problem areas in diabetes (PAID) questionnaire in development of care | Not formally appraised as this is used as a framing for developmentally appropriate care for this group of patients. |
