# Supplementary material for: Living with early-onset type 2 diabetes in young adulthood: a narrative review of lived experience and implications for developmentally appropriate care
Source: Front Endocrinol (Lausanne). 2026 Sep 22;17:1903159. doi: 10.3389/fendo.2026.1903159 (PMC13640145; doi:10.3389/fendo.2026.1903159)
Supplement: Supplementary file 2 [file SupplementaryFile2.docx]

**Supplementary Table S3. Characteristics and key findings of all included studies (n = 35) contributing to the narrative synthesis.**

*This table lists all 35 studies contributing to the narrative synthesis (29 primary studies and 6 review-level sources), consolidating study characteristics and principal findings in one place. It complements Supplementary Tables S2A and S2B, which report the design-appropriate methodological appraisal of the same studies, and Table 2, which highlights selected quantitative indicators for rapid clinical appraisal. Evidence classification follows the direct / closely related supporting / contextual hierarchy defined in the Methods.*

**Primary studies (n = 29)**

| **Study (Author, Year, Country) [Ref.]** | **Study design** | **Sample size & age range** | **Evidence classification** | **Domain(s) examined** | **Principal finding(s)** |
| --- | --- | --- | --- | --- | --- |
| Hessler DM et al., 2011- USA (California/Colorado; per author affiliations, verified by literature search, not stated in provided materials) [11] | Cross-sectional (JBI analytical) | n=506 adults with T2DM, aged 20 to 80 years; community/clinical sample | Contextual (broader adult T2DM population) | Diabetes distress, self-efficacy, self-management, HbA1c, by age | Younger age was associated with greater diabetes distress (β=−0.21, p<0.001), lower self-efficacy (β=0.18, p<0.001), poorer diet (β=0.20, p<0.001) and higher HbA1c. Stress and distress were associated with higher HbA1c in younger but not older adults. |
| Trief PM et al., 2022 - USA (multi-site TODAY2 cohort) [12] | Cross-sectional (JBI analytical); iCount sub-study of TODAY2 follow-up | n=572, mean age ≈26 years; youth-onset T2DM (diagnosed <18 years) | Closely related supporting (youth-onset, not adult-onset T2DM) | Medication adherence | 69.8% prescribed oral glucose-lowering medication and 37.0% prescribed insulin took <80% of prescribed treatment. Lower adherence was associated with negative medication beliefs, lower self-efficacy, diabetes distress, food insecurity and unmet material needs. |
| Sim RRJ et al., 2024 - Singapore [18] | Qualitative (CASP) | n=21, aged 22–39 years; three Singapore polyclinics | Direct | Stigma; self-management education preferences; healthcare communication | Stigma arising from self, others and healthcare professionals could discourage treatment-related behaviours (e.g. medication-taking); medical jargon reduced understanding. Participants valued age-peer contact and flexible/remote appointments compatible with work and caregiving. |
| Kneck A et al., 2012 - Sweden [19] | Qualitative (CASP) | n=13, diagnosed 3 years previously; aged 20–80 years | Contextual (broad age range, not specific to early-onset) | Illness meaning; self-management | Living with diabetes described as a continuous process of balancing, adaptation and interpretation of bodily symptoms; uncertainty about bodily needs increased dependence on others; diabetes described as never fully “mastered.” |
| Gopalan A et al., 2022 - USA (California) [21] | Qualitative (CASP) | n=41, aged 21–44 years, diagnosed within prior 2 years; integrated California health system | Direct (upper age 44 slightly exceeds 39) | Family experience; illness meaning | Family exposure to diabetes increased perceived seriousness and motivated self-care for some, while normalising the condition and fostering fatalism for others. Parenthood could motivate healthier behaviours while caregiving reduced time/energy for self-management. |
| Bień A et al., 2016 - Poland [23] | Cross-sectional (JBI analytical) | Pregnant women with diabetes; not restricted to early-onset T2DM | Contextual | Diabetes acceptance / quality of life (pregnancy context) | More adaptive diabetes-identity integration/acceptance was associated with better psychosocial functioning and self-management (cited alongside refs 24, 25 for this association). |
| Celik A et al., 2023 - UK and Turkey [24] | Qualitative (CASP) | n=36 women, aged 20–45 years | Direct (upper age 45 slightly exceeds 39) | Reproductive concerns; healthcare communication | Young women described anxiety about fertility, pregnancy-related risk and their children's future health; these concerns were often not discussed with healthcare professionals, leaving uncertainty unsupported. |
| Schmitt A et al., 2014 - Germany [25] | Cross-sectional (JBI analytical) | n=320 mixed diabetes clinical sample (96 with T2DM); not specific to young-onset T2DM | Contextual | Diabetes acceptance, self-care, glycaemic control | Cited (with refs 22, 24) for the association between negative diabetes identity/stigma and concealment, avoidance, distress and impaired self-care. |
| Wong SKW et al., 2024 (qualitative) - Singapore [27] | Qualitative (CASP) | n=21, aged 22–39 years; Singapore polyclinics | Direct | Stigma; self-care patterns; peer relationships | Stigma and self-blame led some participants to conceal their diagnosis or avoid revealing behaviours. Four self-care mindset patterns identified (avoidant, indifferent, striving, activated), with lowest self-care in avoidant/indifferent groups. Peer relationships had both supportive and detrimental effects. |
| Ke C et al., 2019 - Hong Kong [28] | Cohort (JBI cohort) | Large population-based young-onset T2DM cohort (36.8% early-onset) | Closely related supporting (young-onset cohort; comparison by age of onset) | Mental-health hospitalisation | Among those aged 20–39, mental illness accounted for 28% of hospitalisations (587.2 bed-days/1,000 patient-years); 36.8% of bed-days before age 40 were attributable to mental illness (55.1% psychotic disorders, 31.4% mood disorders). |
| Eitel KB et al., 2023 - USA (SEARCH, multicentre) [29] | Cross-sectional (JBI analytical) | n=1,608 (SEARCH cohort); 353 (22%) youth-onset T2DM | Closely related supporting | Stigma; clinical outcomes | Higher diabetes stigma scores were associated with insulin use and retinopathy. |
| Holmes-Truscott E et al., 2020 - Australia (MILES-2) [30] | Cross-sectional (JBI analytical) | Large national Australian survey (n=2,088); 54% T2DM, broader age distribution | Contextual | Stigma; psychological wellbeing | Diabetes stigma was associated with poorer psychological wellbeing (greater depressive symptoms, anxiety, diabetes distress); self-esteem and social support partly mitigated this impact. |
| Wong SKW et al., 2024 (quantitative) - Singapore [31] | Cross-sectional (JBI analytical) | n=97 early-onset vs n=312 usual-onset T2DM | Direct | Self-efficacy; diabetes knowledge | Similar diabetes knowledge but significantly lower self-efficacy in early-onset vs usual-onset T2DM (adjusted B=−0.19, 95% CI −0.35 to −0.03). |
| He Q et al., 2023 - China [32] | Cross-sectional (JBI analytical) | n=227, aged 18–44 years; two Chinese hospitals | Direct (upper age 44 slightly exceeds 39) | Self-management behaviours; peer/digital support | Self-care was lowest in foot care and blood-glucose testing, highest in medication-taking; dietary control and glucose testing declined with longer duration. WeChat group participation was positively associated with exercise, treatment discussion and dietary choices; some found support groups poorly tailored to age/life stage. |
| Zhao X et al., 2025 - UK [32] | Qualitative (CASP) | Purposive sample, aged 18–40 years; young-onset T2DM | Direct | Physical activity barriers/facilitators | Work/family responsibilities, weight-related concerns and stigma limited physical activity; social support, shared activity and self-monitoring facilitated engagement. |
| Britton LE et al., 2023 - USA [34] | Qualitative (CASP) | n=32 Black and/or Latina women, aged 18–40 years | Direct | Caregiving and self-management | Caregiving could motivate women to remain healthy for their children/families while simultaneously competing with self-management for time, energy and financial resources — a bidirectional influence. |
| Bower P et al., 2023 - UK (Greater Manchester) [35] | Mixed-methods (MMAT); Q-methodology + qualitative | n=43 (of 46 completing Q-sort), aged 18–40 years | Direct | Patient perspectives / barriers-facilitators typology | Identified five distinct perspectives: stressed/coping with crises; financially disadvantaged/poorly supported; well-intentioned but struggling; withdrawn/worried; and particularly affected by the stigma of diabetes at a young age. |
| Misra S et al., 2023 - UK [36] | Cross-sectional (JBI analytical); National Diabetes Audit | n=122,780; 98% aged 18–39 years | Direct | Socioeconomic disadvantage; glycaemic/cardiovascular risk | Younger adults were disproportionately represented in the most deprived quintiles (35% vs 29% aged 40–59). Greater deprivation was associated with higher HbA1c, albuminuria and predicted cardiovascular risk. |
| Wijayaratna S et al., 2021 - New Zealand (Auckland) [37] | Cross-sectional (JBI analytical) | Young people diagnosed aged 15–30 years; T1DM/T2DM mixed | Closely related supporting (includes <18 at diagnosis; mixed diabetes types) | Medication access | Only 63% had diabetes medication dispensed; nearly one-third with severe hyperglycaemia had received no diabetes medication in the preceding 6 months. |
| Malik FS et al., 2023 - USA (SEARCH) [38] | Cross-sectional (JBI analytical) | n=2,561 young adults (384 with T2DM), multicentre | Closely related supporting (youth-onset SEARCH cohort) | Food insecurity | Household food insecurity was reported in 30.7% of participants with T2DM vs 17.7% with T1DM. Lower income, lower parental education and lack of insurance were independently associated with greater odds of food insecurity. |
| Reid LA et al., 2022 - USA (SEARCH) [39] | Cross-sectional (JBI analytical) | Youth/young adults with youth-onset T2DM, multicentre | Closely related supporting | Food insecurity; diabetic ketoacidosis | Household food insecurity was associated with 3.08-fold higher odds of diabetic ketoacidosis compared with food security. |
| Gucciardi E et al., 2009 - Canada [40] | Cross-sectional (JBI analytical) | Large Canadian population survey; broad age range | Contextual | Food insecurity | Each 1-year younger age at T2DM diagnosis was associated with a 4% increase in the odds of household food insecurity (OR 1.04, 95% CI 1.02–1.05). |
| Nagata JM et al., 2019 - USA [41] | Cross-sectional (JBI analytical) | Nationally representative US young-adult cohort | Contextual | Food insecurity; chronic disease | Food insecurity was associated with poorer self-reported health status (OR 2.63, 95% CI 1.63–4.24) and with diabetes (OR 1.67, 95% CI 1.18–2.37). |
| Weinstein JM et al., 2022 - USA [42] | Cross-sectional (JBI analytical) | Large nationally representative working-age US adults with diabetes | Contextual | Food insecurity; healthcare utilisation | Food insecurity was associated with more than twice the number of missed workdays among working adults with diabetes. |
| Tschirhart H et al., 2024 – Canada [44] | Qualitative (CASP) | n=18 pregnant women; pre-existing T1DM/T2DM (mixed), specialist-clinic sample | Closely related / contextual (mixed diabetes types) | Diabetes distress in pregnancy | Pregnancy intensified monitoring, treatment demands and uncertainty about fetal/maternal outcomes, increasing burden and undermining self-efficacy. |
| Sidhu T et al., 2022 - UK [47] | Qualitative (CASP) | n=20 (12 South Asian, 8 White), median age 60.2 years; 60% T2DM | Contextual (median age 60; broader T2DM/sociocultural context) | Gendered expectations; physical activity; sociocultural norms | Gendered expectations restricted physical activity in some communities. |
| Singh H et al., 2012 - UK [48] | Qualitative (CASP) | n=20 outpatients (12 British South Asian, 8 White); one UK hospital | Contextual | Sociocultural stigma; support systems | Diabetes and body weight could intensify stigma in some communities where these affect perceptions of social or marital suitability. |
| Savage S et al., 2009 - Australia [49] | Qualitative (CASP) | n=13, aged 25–45 years | Direct (upper age 45 slightly exceeds 39) | Education and support preferences | Existing education/support programmes were perceived as oriented towards older adults and insufficiently responsive to younger adults' concerns; participants wanted practical, usable, consistent, age-appropriate advice and greater involvement in self-management decisions; life-stage issues (pregnancy, surgery) not always addressed; digital-platform convenience did not necessarily ensure relevance or credibility. |
| Rasmussen B et al., 2016 - Australia and Denmark [50] | Qualitative (CASP) | n=26 (12 Australia, 14 Denmark), aged 19–42 years; T2DM duration >10 months | Direct (upper age 42 slightly exceeds 39) | Psychosocial life-transitions; education preferences | Young adults wanted practical, usable, consistent, age-appropriate advice and greater involvement in self-management decisions (cited jointly with Savage et al. above). |

**Review-level sources (n = 6)**

| **Study (Author, Year, Country) [Ref.]** | **Study design** | **Sample size & age range** | **Evidence classification** | **Domain(s) examined** | **Principal finding(s)** |
| --- | --- | --- | --- | --- | --- |
| Lake AJ et al., 2018 - review (N/A) [20] | Narrative review (SANRA) | N/A — review-level synthesis | Supporting (review-level; directly focused on adults 18–39 years with T2DM) | Perceived complication risk; retinal-screening engagement | Low perceived susceptibility to diabetic retinopathy, especially without symptoms, was identified as a barrier to screening uptake; complications perceived as distant may be given lower priority in young adulthood. |
| Hopkins L et al., 2025 - review (N/A) [43] | Systematic review of qualitative studies (JBI) | N/A — review-level synthesis | Supporting (reproductive-care evidence, not restricted to 18–39 years) | Reproductive/pregnancy care experience | Identified tension between personal reproductive goals and the need to optimise diabetes management pre-conception; care across pre-pregnancy, pregnancy and postpartum periods described as fragmented, with unclear specialist pathways and disjointed transitions. |
| Patil SJ et al., 2016 - review (N/A) [45] | Systematic review / meta-analysis (JBI) | N/A — review-level synthesis | Contextual (general adult diabetes population) | Peer support interventions | Peer support interventions were associated with a small but statistically significant reduction in HbA1c of 0.24% (95% CI 0.05–0.43). |
| Li-Geng T et al., 2020 - review (N/A) [46] | Mixed-methods systematic review (JBI) | N/A — review-level synthesis | Contextual (East Asian American adults; older/general adult population) | Sociocultural/dietary self-management | Dietary choices were shaped by beliefs about food, family roles and maintaining social harmony; following a separate diabetes diet could reduce enjoyment of shared meals, create family tension or feelings of burdensomeness. |
| Ramadas A et al., 2011 - review (N/A) [52] | Systematic review (JBI) | N/A — review-level synthesis | Contextual (general adult T2DM; dated evidence base) | Digital/web-based interventions | Supports features such as personalised feedback, goal-setting and interaction in digital diabetes education. |
| Gabarron E et al., 2018 - review (N/A) [53] | Rapid evidence review (JBI, rapid-review caveat) | N/A — review-level synthesis | Contextual | Social media/digital interventions | Convenience of digital platforms does not necessarily ensure relevance or credibility; supports centralised, credible resources rather than simply transferring existing educational material online. |

Abbreviations: CASP, Critical Appraisal Skills Programme; JBI, Joanna Briggs Institute; MMAT, Mixed Methods Appraisal Tool; SANRA, Scale for the Assessment of Narrative Review Articles; T1DM, type 1 diabetes mellitus; T2DM, type 2 diabetes mellitus; HFI, household food insecurity; DKA, diabetic ketoacidosis; N/A, not applicable.
